# Potency, selectivity and prolonged binding of saxagliptin to DPP4: maintenance of DPP4 inhibition by saxagliptin in vitro and ex vivo when compared to a rapidly-dissociating DPP4 inhibitor

**DOI:** 10.1186/1471-2210-12-2

**Published:** 2012-04-04

**Authors:** Aiying Wang, Charles Dorso, Lisa Kopcho, Gregory Locke, Robert Langish, Eric Harstad, Petia Shipkova, Jovita Marcinkeviciene, Lawrence Hamann, Mark S Kirby

**Affiliations:** 1 Departments of Metabolic Diseases - Diabetes, Pennington NJ 08534, USA; 2 Chemical Enzymology, Pennington NJ 08534, USA; 3 Bioanalytical and Discovery Analytical Sciences, Pennington NJ 08534, USA; 4 Drug Safety Evaluation and, Pennington NJ 08534, USA; 5 Chemistry, Research and Development, Bristol-Myers Squibb Company, 311 Pennington-Rocky Hill Road, Pennington NJ 08534, USA

## Abstract

**Background:**

Dipeptidylpeptidase 4 (DPP4) inhibitors have clinical benefit in patients with type 2 diabetes mellitus by increasing levels of glucose-lowering incretin hormones, such as glucagon-like peptide -1 (GLP-1), a peptide with a short half life that is secreted for approximately 1 hour following a meal. Since drugs with prolonged binding to their target have been shown to maximize pharmacodynamic effects while minimizing drug levels, we developed a time-dependent inhibitor that has a half-life for dissociation from DPP4 close to the duration of the first phase of GLP-1 release.

**Results:**

Saxagliptin and its active metabolite (5-hydroxysaxagliptin) are potent inhibitors of human DPP4 with prolonged dissociation from its active site (Ki = 1.3 nM and 2.6 nM, t_1/2 _= 50 and 23 minutes respectively at 37°C). In comparison, both vildagliptin (3.5 minutes) and sitagliptin ( < 2 minutes) rapidly dissociated from DPP4 at 37°C. Saxagliptin and 5-hydroxysaxagliptin are selective for inhibition of DPP4 versus other DPP family members and a large panel of other proteases, and have similar potency and efficacy across multiple species.

Inhibition of plasma DPP activity is used as a biomarker in animal models and clinical trials. However, most DPP4 inhibitors are competitive with substrate and rapidly dissociate from DPP4; therefore, the type of substrate, volume of addition and final concentration of substrate in these assays can change measured inhibition. We show that unlike a rapidly dissociating DPP4 inhibitor, inhibition of plasma DPP activity by saxagliptin and 5-hydroxysaxagliptin in an ex vivo assay was not dependent on substrate concentration when substrate was added rapidly because saxagliptin and 5-hydroxysaxagliptin dissociate slowly from DPP4, once bound. We also show that substrate concentration was important for rapidly dissociating DPP4 inhibitors.

**Conclusions:**

Saxagliptin and its active metabolite are potent, selective inhibitors of DPP4, with prolonged dissociation from its active site. They also demonstrate prolonged inhibition of plasma DPP4 ex vivo in animal models, which implies that saxagliptin and 5-hydroxysaxagliptin would continue to inhibit DPP4 during rapid increases in substrates in vivo.

## Background

Diabetes is a worldwide epidemic, with the World Health organization estimating that more than 220 million people have diabetes worldwide http://www.who.int/mediacentre/factsheets/fs312/en/index.html, with greater than 90% of those having type 2 diabetes mellitus (T2DM). T2DM is thought to develop as a combination of insulin resistance and pancreatic β-cell failure [1]. Therefore, identification of novel treatments that would increase pancreatic insulin secretion while protecting pancreatic β-cells are of great interest.

Incretin hormones, such as glucagon-like peptide-1 (GLP-1), are secreted from cells in the gastrointestinal (GI) tract into the circulation in response to nutrient absorption. They are a major component of the mechanism regulating post-prandial insulin secretion when it is needed following meals [2]. Incretins account for up to 60% of the post-prandial insulin secretion in healthy individuals, but the incretin response is impaired in T2DM [3]. Incretin effects do not lead to insulin release per se, but potentiate the physiological release of insulin from the pancreas in response to increases in plasma glucose. Since GLP-1 has been shown to have the major incretin effect on glucose homeostasis in patients with type 2 diabetes [4], much work has been done to understand the effects of this incretin hormone on normal and pathophysiological glucose homeostasis.

Following its secretion, dipeptidylpeptidase-4 (DPP4) rapidly metabolizes the intact form of GLP-1 (GLP-1_7-36_) to inactive GLP-1_9-36 _with a half-life of 1 to 2 minutes in vivo [5]. Therefore, two approaches have been taken to increase activity of the incretin axis, parenteral administration of DPP4-resistant GLP-1 analogues or oral administration of DPP4 inhibitors. DPP4 inhibitors have minimal risk of hypoglycemia because they enhance glucose-dependent insulin secretion and glucagon reduction. They are also weight neutral; i.e., they do not promote weight gain that is typically seen with many other anti-diabetic agents. DPP4 inhibitors are also effective in combination with several other diabetes drug classes [6-8]. Finally, data from animal models indicate that GLP-1 is a trophic factor for β-cells, and potentiating endogenous incretins with DPP4 inhibitors does increase β-cell function and number, thereby contributing to improvement of β-cell function over the long-term [9].

There are many examples of enzyme inhibitors displaying time-dependence (e.g. [10,11]), with several becoming marketed drugs, including members of the DPP4 inhibitor class [12-14]. In many cases, prolonged pharmacodynamic effects on the target enzyme (when compared to the pharmacokinetics of the drug) confers an advantage over rapidly dissociating compounds, because time-dependent drugs typically require lower plasma levels and reduced drug peak-to-trough ratios, reducing the risk of off-target toxicity [11,15]. In humans, peak GLP-1 secretion occurs during the first phase of secretion, which occurs rapidly following a meal, giving a 2- to 3-fold increase that lasts 30 to 60 minutes [3]. This can be followed by a prolonged phase that gives a small increase in GLP-1 levels above fasting levels for up to 2 hours [reviewed in [16]. Therefore, we hypothesized that if a time-dependent inhibitor has a half-life for dissociation close to the duration of the first phase of GLP-1 secretion, the majority of the enzyme-inhibitor complex would not dissociate during the release of GLP-1 and this would maximize the compound's beneficial effects while minimizing plasma drug levels. DPP4 also has many other substrates in vitro, although only a few have been shown to be cleaved by DPP4 in vivo (reviewed in [17]). Therefore, it would be ideal if binding did not extend past the duration of first phase GLP-1 secretion, such that the inhibitor activity would follow its pharmacokinetics for inhibition of cleavage of other substrates of DPP4, should any such substrates have more prolonged in vivo half-lives relative to GLP-1.

Here we describe the inhibitory properties of saxagliptin and its 5-hydroxy metabolite, which are both slow binding DPP4 inhibitors with extended off-rates from DPP4 at 37°C, similar to the duration of the first phase of release of GLP-1 in vivo. We also use the ex vivo plasma DPP assays that are used in preclinical animal models and the clinic as a biomarker for efficacy, to demonstrate how slow binding compounds such as saxagliptin differ from rapidly dissociating DPP4 inhibitors. We show that saxagliptin does not have a dilution artifact or a large dependence on the pseudo-substrate used in the assay, unlike rapidly dissociating DPP4 inhibitors, and we discuss the significance of these findings.

## Results

### Saxagliptin is a potent inhibitor of human DPP4 in vitro irrespective of substrate

We measured the IC_50 _for inhibition of substrates across a range of substrate concentrations (10 μM to 1000 μM, dependent on substrate) that straddled the Km for the pseudo-substrate gly-pro-pNA (Km 180 ± 8 μM, kcat 40 ± 9 s^-1^, room temp, n = 3), and GLP-1 (Km 24 ± 16 μM, kcat 2.9 ± 0.9 s^-1^; room temp, n = 5), then calculated the Ki for inhibition of cleavage by each substrate (Table 1).

**Table 1:** Inhibition of isolated cloned human DPP4 at room temperature

compound	GLP-1 K_i _(nM)	gly-pro-pNA K_i _(nM)
Saxagliptin	0.41 ± 0.1 (7)	0.45 ± 0.1 (5)

Sitagliptin	2.5 ± 0.7 (4) ***	8 ± 1 (5)***

Vildagliptin	1.5 ± 0.5 (4) ***	7 ± 2 (5)***

mean ± standard deviation (number of independent experiments). ***P < 0.001 versus saxagliptin

Each of the DPP4 inhibitors tested were equipotent inhibitors of GLP-1 and gly-pro-pNA, as expected for inhibitors that are competitive with substrate and bind in the active site of DPP4. Therefore, we used gly-pro-pNA as a substrate in subsequent experiments. Saxagliptin was approximately 10-fold more potent than vildagliptin or sitagliptin under these conditions at room temperature.

### Potency and selectivity of saxagliptin and 5-hydroxysaxagliptin for human enzymes in vitro at 37°C

Routine screening was performed at room temperature. However, as DPP4 inhibition in vivo occurs at 37°C, we measured the potency and selectivity of DPP4 inhibitors in vitro at that temperature (Table 2). The Km and turnover rate of gly-pro-pNA pseudo-substrate for DPP4 increased (Km = 209 ± 18 μM; kcat = 67 ± 4 s^-1^, n = 3), as did the Ki values for inhibition of DPP4 by DPP4 inhibitors (Table 2). Saxagliptin was 10-fold more potent than either vildagliptin or sitagliptin at 37°C. Saxagliptin generates an active metabolite in vivo [18], 5-hydroxysaxagliptin; it was 2-fold less potent than saxagliptin.

**Table 2:** Inhibition of isolated, cloned human DPP4, DPP8 and DPP9 at 37°C

	DPP4 K_i _(nM)	DPP8 K_i _(nM)	DPP9 K_i _(nM)
Saxagliptin	1.3 ± 0.3 (12)	508 ± 174 (13)	98 ± 44 (11)

5-hydroxysaxagliptin	2.6 ± 1.0 (12)***	2495 ± 727 (14)*	423 ± 64 (12)

Vildagliptin	13 ± 2.8 (12)***	5218 ± 2319 (14)***	258 ± 93 (12)

Sitagliptin	18 ± 1.6 (12)***	33780 ± 5532 (12)***	55142 ± 19414 (11)***

mean ± standard deviation (number of independent experiments). *P < 0.05, **P < 0.01, ***P < 0.001 versus saxagliptin

The gly-pro-pNA pseudo-substrate is not specific to DPP4 and is also cleaved by other enzymes, including DPP8 (Km = 792 ± 60 μM; kcat = 5.1 ± 0.4 s^-1^, n = 3) and DPP9 (Km = 221 ± 27 μM; kcat = 3.7 ± 0.7 s^-1^, n = 3), although the cleavage rate of both enzymes is 20-fold lower than DPP4. Therefore, we also used this substrate to investigate inhibition of DPP8 and DPP9 by DPP4 inhibitors. Saxagliptin is approximately 400-fold selective and 75-fold selective for DPP4 versus DPP8 and DPP9, respectively, with the 5-hydroxymetabolite having approximately twice the selectivity (DPP8 approximately 950-fold and DPP9 approximately 160-fold). In comparison, vildagliptin had 400-fold selectivity for DPP8 and 20-fold selectivity for DPP9, while sitagliptin had 1900-fold selectivity for DPP8 and 3000-fold selectivity for DPP9.

Saxagliptin and 5-hydroxysaxagliptin were tested against multiple other enzymes (at room temperature). Both compounds had > 1000-fold selectivity against FAP, and > 6000-fold selectivity against DPP2 and all other proteases tested: these included neutral endopeptidase, angiotensin converting enzyme, aminopeptidase P, prolidase, prolyl carboxypeptidase, activated protein C, chymotrypsin, factor IXa, Factor VIIa, Factor Xa, Factor XIa, factor XIIa, plasma kallikrein, plasmin, thrombin, tissue kallikrein, tissue plasminogen activator, trypsin and urokinase (data not shown). They also had > 10,000-fold selectivity against a panel of 39 unrelated proteins that included 15 G-protein coupled receptors, 4 nuclear hormone receptors, 6 ion channels, 4 other enzymes and 10 transporters (data not shown).

### Potency and selectivity of saxagliptin and 5-hydroxysaxagliptin for inhibition of cynomolgus monkey DPP enzymes in vitro at 37°C

The potency and selectivity for all 4 compounds for inhibition of cynomolgus monkey (rhesus monkey has the same DPP4 DNA sequence) DPP4, DPP8 and DPP9 is shown in Table 3 and is very similar to that found versus the human enzymes.

**Table 3:** Inhibition of isolated, cloned cynomolgus monkey DPP4, DPP8 and DPP9 at 37°C

	DPP4 K_i _(nM)	DPP8 K_i _(nM)	DPP9 K_i _(nM)
Saxagliptin	1.1 ± 0.2 (14)	390 ± 82 (6)	61 ± 5 (6)

5-hydroxysaxagliptin	2.9 ± 1.1 (13)***	2061 ± 658 (6)***	323 ± 60 (6)*

Vildagliptin	6.8 ± 2.0 (14)***	3692 ± 917 (7)***	125 ± 39 (7)***

Sitagliptin	15.6 ± 3.6 (14)***	21949 ± 17461 (6)***	65757 ± 7966 (6)***

mean ± standard deviation (number of independent experiments). *P < 0.05, **P < 0.01, ***P < 0.001 versus saxagliptin

Similar data were also obtained for mouse and rat enzymes (data not shown). Therefore, we confirmed that the potency and specificity of saxagliptin and its 5-hydroxymetabolite were similar across species in vitro. We did not investigate the effects of DPP4 inhibitors on other peptidases from other species because no effect of saxagliptin and 5-hydroxysaxagliptin were seen on the human proteins tested.

### Saxagliptin and 5-hydroxysaxagliptin are long-acting DPP4 inhibitors in vitro

During the course of initial experiments, we noticed that there was time dependence to inhibition of DPP4 by some DPP4 inhibitors. In order to determine time-dependence, we preincubated DPP4 inhibitors with DPP4 and measured the rate of dissociation of DPP4 inhibitors from DPP4 using an 'infinite dilution' method.

The data in table 4 show that saxagliptin and 5-hydroxysaxagliptin have slow binding when tested at 37°C, with t_1/2 _for dissociation of 50 minutes and 23 minutes, respectively. While vildagliptin shows some evidence of slow binding (t_1/2 _= 3.5 minutes), this was much less pronounced. Sitagliptin showed no time dependence (within the limitations of the experimental protocol at < 2 minutes). The time dependence was only found for inhibition of DPP4 and was not seen during experiments investigating the inhibition of DPP8 and DPP9; therefore, these prolonged effects would only relate to inhibition of cleavage of DPP4 substrates by DPP4.

**Table 4:** On and off rates of DPP4 inhibitors at 37°C

Compound	kon, 10^5 ^M^-1 ^s^-1^	koff, 10^-5 ^s^-1^	t_1/2 _(min.)
**37°C**			

Saxagliptin	4.6 ± 0.6	23 ± 1	50

5-hydroxysaxagliptin	0.7 ± 0.1	50 ± 2	23

Vildagliptin	1.2 ± 0.2	330 ± 30	3.5

Sitagliptin	> 100	> 580	< 2

mean ± standard error. Standard errors for kon were calculated from equations (2), (3) and (4), and for koff are from the fits to equation (5)

### Saxagliptin does not have a dilution artifact in plasma DPP assays in vitro

The ex vivo assay measuring inhibition of plasma DPP4 activity has been used as a key biomarker assay for DPP4 inhibitor assessment by multiple groups in both animal models and in the clinic. Given that the duration of the ex vivo assay is typically between 10 and 20 minutes, there would be no dilution artifact predicted for ex vivo determination of inhibition from saxagliptin dosed animals, because negligible dissociation of saxagliptin from DPP4 would occur over the time frame of the experiment (Table 4 and the rate of dissociation is decreased with lower temperature). To confirm this for saxagliptin, we took naive human plasma and compared samples with just the addition of compound to plasma only, to samples where compound was added to both plasma and buffer (such that total concentration of drug was kept constant during the 'dilution step').

**Figure 1:**
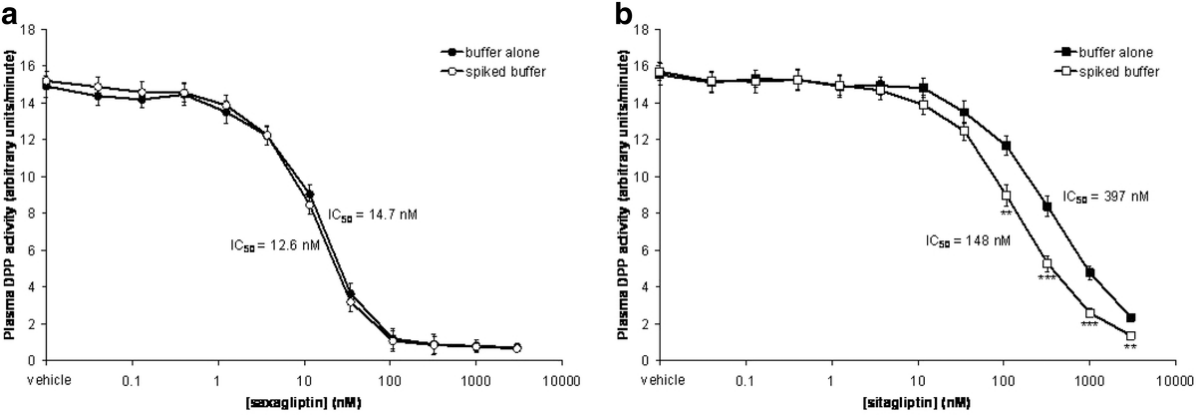
**The effect of dilution on the IC_50 _for inhibition of human plasma DPP activity by saxagliptin (Panel A) and sitagliptin (Panel B) using ala-pro-AFC as substrate (activity is measured as arbitrary fluorescence units**. Mean ± s.e.m., n = 3 independent experiments, 3 replicates per experiment).

Saxagliptin is unaffected by the 3-fold dilution in the human plasma assay (Figure 1a) using ala-pro-AFC as substrate. However, sitagliptin clearly has a dilution artifact (Figure 1b). When sitagliptin was added only to the plasma, the inhibition curve shifted 3-fold to the right compared to when compound was added to both the plasma and the dilution buffer (IC_50 _= 152 ± 41 nM versus 414 ± 116 nM: mean ± s.d., n = 3), except where there is virtually no inhibition or full inhibition, consistent with the 3-fold dilution during substrate addition. This is presumably due to a new equilibrium being rapidly established following dilution, such that the potency of sitagliptin will be underestimated when compound is only present in the plasma. Similar data were also obtained using both cynomolgus and rhesus monkey plasma (data not shown).

### Maximum inhibition of plasma DPP4 activity in plasma samples by DPP4 inhibitors differs between species

**Figure 2:**
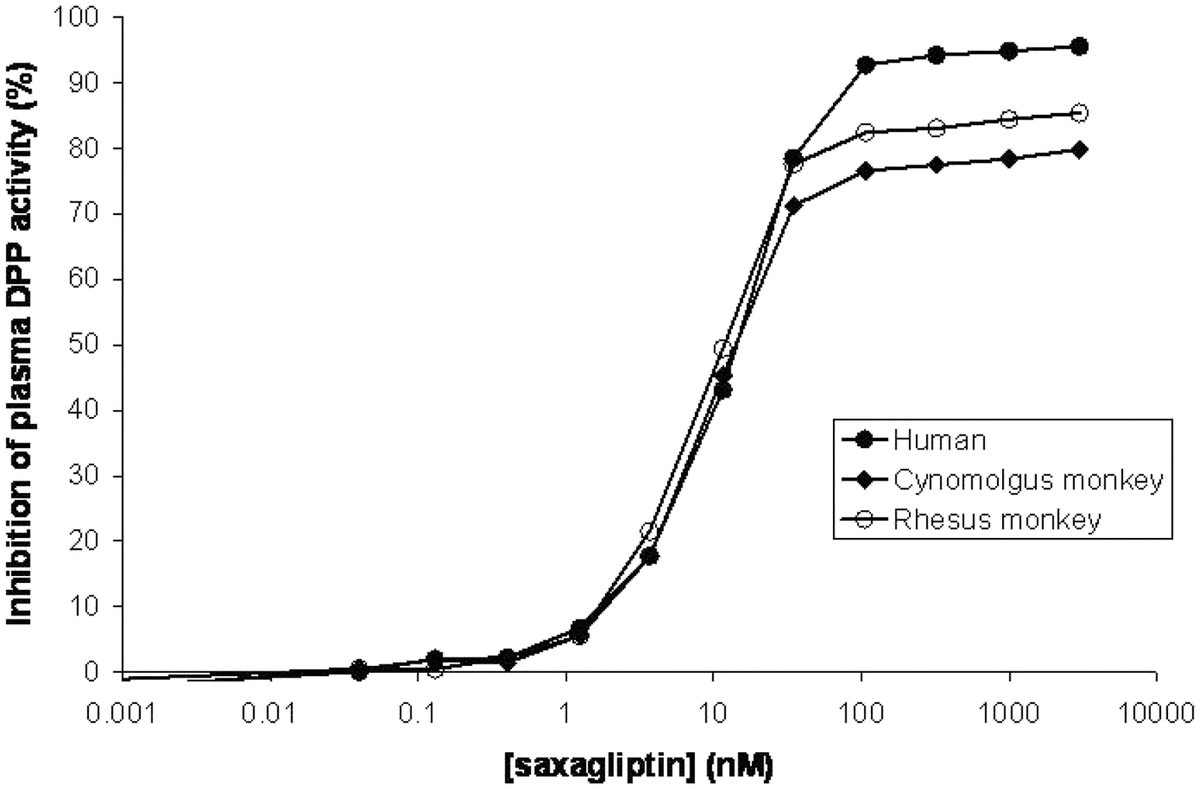
**Percent inhibition of plasma DPP activity in human, rhesus monkey and cynomolgus monkey plasma by saxagliptin using ala-pro-AFC as substrate**.

Untreated human plasma samples gave a plasma DPP enzyme activity rate of 5.0 ± 0.6 nmoles/min per ml plasma (mean ± s.d., n = 3 independent experiments) when ala-pro-AFC was used as substrate. Untreated cynomolgus and rhesus monkey plasma DPP4 rates were similar to those seen in human, with rates of 5.2 ± 0.3 and 7.3 ± 0.2 nmoles/min per ml plasma, respectively. However, the ability of DPP4 inhibitors to inhibit cleaveage of peudo-substrates differs among species. Figure 2 shows that the maximum inhibition of plasma DPP activity seen with saxagliptin was approximately 85% in rhesus and 80% in cynomolgus monkeys, but > 95% in humans (Figure 2). Like human, rodent (mouse and rat) and dog plasma DPP is inhibited > 95% by saxagliptin (data not shown).

**Figure 3:**
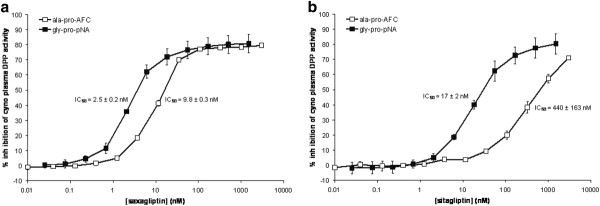
**Inhibition of cynomolgus monkey plasma DPP activity by saxagliptin (Panel A) and sitagliptin (Panel B) using the ala-pro-AFC and gly-pro-pNA assays (mean of 3 independent experiments)**.

These effects were shown to be independent of DPP4 inhibitor and similar data were obtained with gly-pro-pNA as the pseudo-substrate (Table 5; Figure 3). Since the pseudo-substrates are not specific for DPP4, presumably these findings reflect a species difference in the relative activity of all the other plasma peptidases that cleave these pseudo-substrates.

**Table 5:** Maximum levels of inhibition of human and monkey plasma DPP activity using ala-pro-AFC as substrate

	Rhesus		Cyno		Human	
	**IC_50 _(nM)**	**Max % inhib**	**IC_50 _(nM)**	**Max % inhib**	**IC_50 _(nM)**	**Max % inhib**

saxagliptin	2.9 ± 0.3	86 ± 1^†††^	2.4 ± 0.2	81 ± 6^††^	4.0 ± 0.8	100 ± 0

vildagliptin	17 ± 5**	85 ± 1^†††^	13 ± 4**	79 ± 6^††^	20 ± 7*	98 ± 1

sitagliptin	17 ± 2***	87 ± 1^†††^	17 ± 2***	80 ± 7^††^	22 ± 3***	98 ± 0

N = 3 independent experiments. *P < 0.05, **P < 0.01, ***P < 0.001 versus saxagliptin. ^††^P < 0.01, ^†††^P < 0.001 versus human

The in vitro IC_50 _for inhibition of human, rhesus monkey and cynomolgus monkey plasma DPP activity by saxagliptin, vildagliptin and sitagliptin was similar across species (Table 5). Therefore, although there were different amounts of maximal inhibition among species, DPP4 inhibitors have similar potency in rhesus monkey, cynomolgus monkey and human plasma for inhibition of plasma DPP activity.

### Choice of assay affects the IC_50 _measured for inhibition of plasma DPP activity by DPP4 inhibitors at steady-state in vitro

The measured IC_50 _varies with the ratio of substrate concentration to substrate Km for competitive inhibitors (see Methods). Using the assays described here, Km values were calculated as 57 ± 13 μM (n = 3 independent experiments) for the ala-pro-AFC assay, and 180 ± 10 μM (n = 3) for gly-pro-pNA assay in human plasma. Km values were similar in cynomolgus monkey plasma, at 35 ± 6 μM (n = 3) for ala-pro-AFC assay and 134 ± 5 μM (n = 3) for gly-pro-pNA assay. Since the majority of DPP4 inhibitors are competitive with substrate, a difference in substrate concentration will affect the measured IC_50 _of these inhibitors. In the two pseudo-substrate assays we used to measure inhibition of plasma DPP activity, the ratio of Km to substrate concentration is approximately 7-fold in the ala-pro-AFC assay (370 μM substrate concentration), but is only 2-fold in the gly-pro-pNA assay (400 μM substrate concentration). Therefore, using gly-pro-pNA as substrate would give an apparent increase in potency of DPP4 inhibitors compared to ala-pro-AFC. Further, the difference in temperature (30°C versus room temperature) and pH (7.4 versus 7.9) of the two assays would also affect the measured IC_50_. Figure 3 shows the inhibition of cynomolgus monkey plasma DPP activity in vitro for saxagliptin and sitagliptin under pseudo-steady-state conditions. There was a small change in IC_50 _for saxagliptin (2.5 ± 0.2 nM to 9.8 ± 0.3 nM, P < 0.0001), with a narrow concentration range over which a difference would be seen between the two assays (1 to 10 nM. Figure 3A). This presumably reflects differences in temperature and pH between the two assays. However, at concentrations of sitagliptin between 5 and > 3000 nM, much more inhibition of DPP activity is seen with the gly-pro-pNA assay than with the ala-pro-AFC assay (Figure 3B). Further, the IC_50 _for inhibition in the ala-pro-AFC assay was significantly increased 26-fold compared to the gly-pro-pNA assay, from 17 ± 2 nM to 440 ± 163 nM (P < 0.0001). Similar data were obtained using human plasma (data not shown).

### Measurement of plasma DPP activity in ex vivo assays

**Figure 4:**
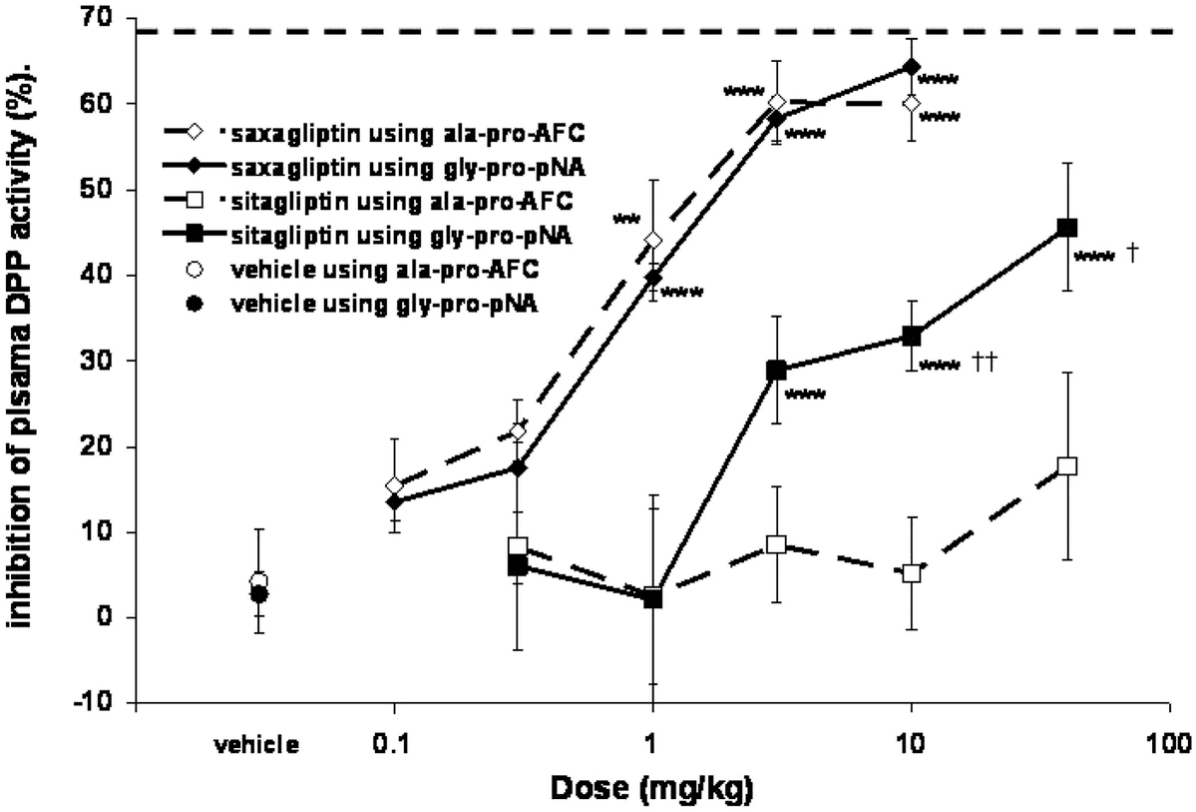
**The effect of pseudo-substrate choice on the measurement of plasma DPP activity ex vivo in cynomolgus monkeys 24 hours post dose**. **P < 0.01, ***P < 0.001 versus vehicle. ^†^P < 0.05, ^††^P < 0.01, versus ala-pro-AFC at the same dose. The dashed line represents the average maximum inhibition seen (68.9 ± 3.9%, 1 hour post dose at the highest doses tested).

The differences in dissociation rate from DPP4 and the substrate used have substantial implications for measurement of activity following dosing in animals and humans. Figure 4 shows data from an in vivo study where various doses of saxagliptin and sitagliptin were given to cynomolgus monkeys and plasma DPP inhibition was measured after 24 hours, at trough.

When the ala-pro-AFC assay was used to measure plasma DPP inhibition ex vivo, saxagliptin treatment resulted in close to maximal inhibition of the inhibitable plasma DPP activity at its highest doses, with the 1, 3 and 10 mg/kg doses being statistically different from vehicle. However, sitagliptin treatment had no effect on plasma DPP activity at any of the doses. When the gly-pro-pNA assay was run on exactly the same samples, similar results were obtained for saxagliptin and there was no statistical difference between the data obtained with either assay at any dose. In contrast to the ala-pro-AFC assay, sitagliptin treatment gave statistically significant inhibition of plasma DPP activity at 3, 10 and 40 mg/kg doses when compared to vehicle in the gly-pro-pNA assay. Further, plasma DPP inhibition in the gly-pro-pNA assay at the 10 and 40 mg/kg doses were statistically significantly different from those obtained using the ala-pro-AFC assay (P = 0.07 for the 3 mg/kg dose). However, the highest dose tested still did not give maximal inhibition of plasma DPP activity. Therefore, choice of assay had significant relevance for the interpretation of inhibition by sitagliptin in this study.

## Discussion

Saxagliptin (BMS-477118) is a potent inhibitor of DPP4 that is approximately 10-fold more potent than vildagliptin or sitagliptin. Saxagliptin also has an active metabolite, 5-hydroxysaxagliptin, which is present in human plasma at levels between 2- and 7-fold higher than saxagliptin (Fura 2009). 5-hydroxysaxagliptin is 2-fold less potent than saxagliptin, but approximately 5-fold more potent than sitagliptin and vildagliptin.

The data here shows that temperature and the choice of substrate can have significant effects on IC_50 _values, accounting for some of the differences in values typically reported (reviewed in [17]). However, Thomas et al. [14] reported very high IC_50 _values for saxagliptin and vildagliptin in particular. In the case of saxagliptin, the value was 50-fold higher than the Ki value reported here and 50-fold higher than the value reported for their DPP4 inhibitor, linagliptin. Saxagliptin (Onglyza^®^) is available commercially in many world markets at doses of 2.5 mg and 5 mg [6] and the minimal efficacious dose of linagliptin is 5 mg [19]. It is difficult to conceive how saxagliptin could be 50-fold less potent than linagliptin in vitro, but at least equipotent in the clinic; therefore, it seems likely that the data reported here are more accurate determinations of the activity of saxagliptin and vildagliptin. A known concentration of pure DPP4 is typically used in the majority of these in vitro studies (we used 100 pM of isolated cloned human DPP4) because a low concentration of DPP4 is important for avoiding obtaining artifactually high inhibition values. Part of the reason for the discrepancy may be that Thomas et al. [14] are the only group to use "DPP4 extracted from Caco cell membranes", with DPP4 purity and concentration undisclosed.

The increased potency and prolonged binding of saxagliptin compared to sitagliptin and vildagliptin may be a reflection of its strong interactions with both Ser^630 ^and Glu^205^/Glu^206^, whereas sitagliptin interacts primarily with Glu^205^/Glu^206^, and vildagliptin with Ser^630 ^[17]. Further, we showed that saxagliptin demonstrated slow-onset inhibition at room temperature that was partially due to a covalent, but reversible enzyme-adduct formation at Ser^630^, but also partially due to another conformational change induced by saxagliptin [20]. Both saxagliptin and 5-hydroxysaxagliptin also have slow binding kinetics at 37°C and would be expected to have prolonged pharmacodynamic effects in vivo.

The data in Figure 4 shows that saxagliptin bound to DPP4 prior to substrate addition remained bound during the time course of the ex vivo assay. The half life for dissociation of saxagliptin from DPP4 (t_1/2 _of 50 minutes at 37°C) is similar to the duration of first phase of elevated GLP-1 in the plasma following a meal in humans [4]. This may be relevant to the cleavage of endogenous substrate(s) because saxagliptin (and 5-hydroxysaxagliptin) given before a meal will already be bound to DPP4 at its site(s) of action. When incretins are released in response to a meal, incretins will increase and compete with inhibitors with rapid off-rates. However, this would be unlikely to occur for saxagliptin, unless substrate concentrations were raised for significantly longer than the duration of the first phase of GLP-1 secretion, when saxagliptin would equilibrate with substrate as with the other DPP4 inhibitors. In clinical practice, DPP4 inhibitors currently have a placebo-like side-effect profile in trials up to 2 years in length. Therefore, rapidly dissociating DPP4 inhibitors can be given at high enough doses that any theoretical advantages from extended binding are currently not seen in the clinic. However, the chronic adverse event profiles of current DPP4 inhibitors are not yet fully defined and differences between DPP4 inhibitors may yet be seen.

Saxagliptin and 5-hydroxysaxagliptin are selective for DPP4 versus DPP8 and DPP9 at 37°C. Neither saxagliptin nor 5-hydroxysaxagliptin exhibited slow binding kinetics to DPP8 or DPP9, so any DPP8/9 related pharmacodynamics would closely match their pharmacokinetics. The potential for inhibition of DPP8 and DPP9 in vivo is difficult to assess in the absence of a known physiologically relevant substrate and knowledge of the specific tissues and cells where either may play a physiological role. Further, as DPP8 and DPP9 are cytosolic enzymes [21], the cytosolic concentration of saxagliptin and 5-hydroxysaxagliptin in those cells would also be required to accurately predict the potential for inhibition. Since this information is unknown (and to test for other off-target issues), extensive toxicity studies are typically undertaken in several species and adverse events are scrutinized in clinical studies. As discussed previously (reviewed in [17]), the preponderance of evidence for saxagliptin would show that there is no toxicity attributable to inhibition of DPP8 or DPP9 at clinically relevant doses across species.

There was no significant species difference for potency of inhibition of human and cynomolgus monkey DPP4, DPP8, DPP9 or plasma DPP activity for three DPP4 inhibitors (saxagliptin, vildagliptin or sitagliptin). However, there were significant differences in the maximum amount of inhibition of plasma DPP activity seen between rhesus and cynomolgus monkey plasma (80 to 85%) and the other species tested (95%). This presumably shows a difference between the levels of dipeptidylpeptidases found in the plasma of the two species. The identities of the peptidases that underlie this difference are unknown and were not a focus of these experiments. However, as there was no differentiation between saxagliptin, vildagliptin and sitagliptin, it is unlikely to be attributable to species differences in DPP8 or DPP9 expression (also consistent with current understanding that DPP8 and DPP9 are not secreted or thought to be present in plasma [17]).

Based on the data presented here, interpretation of data from ex vivo assays for DPP4 inhibitors should be done with caution because the choice of substrate, as well as the nature of the ex vivo assay (such as dilution artifact), can all affect measured efficacy. The difference in plasma DPP inhibition for sitagliptin between these two assays (Figure 4) can be explained by sitagliptin's rapid dissociation and reassociation with the active site of DPP4. Due to the difference in the ratio of Km to substrate concentration between the two assays, one substrate is less effective at competing out the inhibitor and the inhibitor appears more potent in that assay. Consistent with this conclusion was the in vitro data that showed that this issue would most likely manifest itself across the range of plasma concentrations typically found at trough (24-hour post dose) for sitagliptin in cynomolgus monkeys. In contrast, saxagliptin would not dissociate appreciably from the active site during the time course of the assay and there would be no inhibitor-substrate competition.

Krishna et al. [22] recently illustrated the benefits of accelerating drug development using biomarkers with the example of DPP4 inhibitors and the plasma DPP4 assay. While this approach can be fundamentally useful, some important caveats to this approach have been demonstrated here. The pseudo-substrate gly-pro-pNA was used in both clinical assays to determine plasma DPP inhibition for saxagliptin and sitagliptin. However, the conditions are very different: i.e. the substrate concentration is 2000 μM (10-fold the Km), with an 11-fold dilution step and 120 minute assay duration in the saxagliptin assay [23]; the substrate concentration is 400 μM (2-fold Km), with a 2.5-fold dilution step and a 14 minute assay duration in the sitagliptin assay [24]. The data presented here show that, given the comparably longer duration of the assay, the larger dilution factor and the higher substrate concentration in the saxagliptin clinical assay, it is not possible to perform a meaningful direct comparison between clinical data for the two drugs using these different assays. This may explain why Krishna et al. [22] reported that they need 80% inhibition of plasma DPP4 activity at trough (24-hours post dose) to obtain maximal glucose lowering for sitagliptin. However, only 50% to 60% inhibition of plasma DPP activity was seen 24-hours post dose for vildagliptin at the 100 mg dose [25] and for saxagliptin at the 5 mg dose [23]. Despite these differences, all the DPP4 inhibitors have similar glucose-lowering efficacy in the clinic at those doses, leading Neumiller et al. [6] to conclude that "there does not appear to be a compelling advantage of one DPP-4 inhibitor over another".

Further, saxagliptin is localized to the GI tract when given intra-arterially in animal models [18]. Holst and Deacon [26] proposed that GLP-1 cleavage in the GI tract is important: therefore, saxagliptin, by virtue of its location in the GI tract, may give rise to total inhibition of GLP-1 cleavage, while cleavage of another substrate in a different tissue could be unaffected, and the ex vivo plasma DPP assay shows only partial inhibition of DPP4. Further, the tissue distribution of all DPP4 inhibitors is unlikely to be the same and different DPP4 inhibitors may have different effects if there is tissue-specific localization of inhibitor or substrate. Thus, the action of a DPP4 inhibitor in the relevant tissue can only be approximated by the evaluation of ex vivo plasma DPP activity.

## Conclusions

Saxagliptin and its active metabolite are potent, selective inhibitors of DPP4, with prolonged dissociation from its active site. They also demonstrate prolonged inhibition of plasma DPP4 ex vivo in animal models, which implies that saxagliptin and 5-hydroxysaxagliptin would continue to inhibit DPP4 during rapid increases in endogenous substrates (such as GLP-1 during its physiologic release). However, this remains to be demonstrated for a physiologic substrate in vivo.

Interpretation of data from ex vivo assays for DPP4 inhibitors should be done with caution because the choice of substrate, as well as the nature of the ex vivo assay (such as dilution artifact), can all affect measured efficacy. Therefore, while ex vivo plasma DPP assays may be useful as a biomarker for a compound, for the reasons discussed in this paper, clinically relevant efficacy, efficacy for endogenous substrates, or relative efficacy across DPP4 inhibitors cannot be predicted solely using a plasma DPP activity assay.

## Methods

### Reagents

Saxagliptin (Onglyza^©^, BMS-477118, or (1 *S*,3 *S*,5 *S*)-2-[(2 *S*)-2-Amino-2-(3-hydroxytricyclo[3.3.1.1^3,7^]dec-1-yl)acetyl]-2-azabicyclo[3.1.0]hexane-3-carbonitrile, monohydrate [13], vildagliptin [27], sitagliptin [28], active and DPP4 cleaved glucagon-like-peptide-1 (GLP-1_7-36 _and GLP-1_9-36_, respectively) were synthesized at Bristol-Myers Squibb. All other chemicals were reagent grade, obtained from Sigma (St Louis, MO). For the in vitro assays, plasma from rhesus and cynomolgus monkeys was obtained frozen from Bioreclamation Inc. (Hicksville, NY). Plasma from human blood was obtained from healthy volunteers within BMS (after obtaining written consent, following the BMS standard operating procedure RD-DIR-003, in accordance with the Helsinki Declaration) by collecting blood into EDTA tubes. These tubes were kept on ice and were centrifuged at 3000 rpm, 4°C for 15 minutes (within 15 minutes of collection). Plasma was aliquoted into polypropylene tubes, quick-frozen in powdered dry ice, and stored at -80°C until the assay was performed.

### Steady-state inhibition of isolated cloned human and cynomolgus monkey DPP4, 8 and 9 in vitro at 37°C

All solutions were preheated to 37°C. Inhibitors were preincubated with enzyme for 40 minutes prior to assay initiation. The assay was initiated by substrate addition and contained: 100 mM Aces, 52 mM Tris, 52 mM ethanolamine, pH 7.4, enzyme, substrate (gly-pro-pNA at either 120 μM or 1000 μM), inhibitor (various concentrations) and 1% DMSO (final volume of 100 μl). Production of p-nitroaniline was measured at 405 nm at 30 second intervals over 40 minutes using a Spectramax 340 plate reader (Molecular Probes, Carlsbad, CA). Softmax Pro software from Molecular Devices (Sunnyvale, CA) was used to obtain the initial slope of the reaction in each well, and the fits were inspected to insure that the reactions were linear to a correlation coefficient of 0.99. An Excel template with XLFit (IDBS, Guildford, U.K.) was used for determination of the IC_50 _for inhibition at each substrate concentration. Three models of enzyme inhibition (competitive, uncompetitive, and non-competitive) were used, but all of the inhibitors in our DPP4 program yielded competitive inhibition against gly-pro-pNA. Therefore, IC_50 _was then converted to K_i _assuming competitive inhibition according to:

(1) $$Ki=\frac{IC50}{\left(1+\frac{S}{Km}\right)}$$

The calculated K_i _values for competitive inhibition at each substrate concentration were averaged and reported. DPP8 and DPP9 were tested under identical conditions. The in vitro assays were also used to determine substrate Km, by varying substrate concentration across an appropriate range. Km was calculated using GraFit software (Erithacus, Surrey U.K.)

### Slow binding kinetics at 37°C for human DPP4

All solutions were preheated to 37°C and the assay was run at 37°C. When slow onset inhibition occurs, final Ki* is a function of the koff and kon (Ki* = koff/kon). Progress of DPP4 inhibition was measured under steady-state conditions (E < < I/(1 + S/Km)), initiating the reaction by adding the enzyme to the substrate and inhibitor mixture. Progress curve analysis was performed as described previously [11] to derive k_obs _values:

(2) $$P=v_{s}t+\frac{\left(v_{i}-v_{s}\right)}{k_{obs}}\left(1-\text{exp}(-k_{obs}t\right)$$

where *P *is reaction product, *v_i _*and v_s _are initial and steady-state velocities, respectively, and k_obs _is the pseudo-first order rate constant for the approach to steady-state. These values were subsequently used to generate k_on_, k_off _and final equilibrium Ki*. In the cases where k_obs _was linearly dependent upon I/(1 + S/Km), k_on _and k_off _were calculated from the slope and intercept of the line:

(3) $$k_{obs}=k_{on}I/\left(1+S/Km\right)+k_{\text{off}}$$

When k_obs _dependence on effective inhibitor concentration was hyperbolic, k_on _and k_off_

$$E+I\overset{k_{1}}{\underset{k_{2}}{⇌}}EI\overset{k_{3}}{\underset{k_{4}}{⇌}}E*I$$

were calculated using following equation according to Scheme 1:

(4) $$k_{obs}=\frac{k_{3}I}{Ki\left(1+\frac{S}{Km}\right)+I}+k_{4}$$

where Ki is k_2_/k_1_, k_on _= k_3_/Ki, k_4 _is k_off _and final Ki* = k_off_/k_on_.

We measured the inhibitor dissociation constant independently using dilution experiments because calculating the inhibitor dissociation constant from the final complex (k_4_) is subject to large errors,. Briefly, enzyme (20 nM) and inhibitor (500 nM) were preincubated for 30 minutes at 37°C to allow complete equilibration, then put through a spin column P-30 (BioRad, Hercules, CA) to eliminate excess free inhibitor. The resulting solution was immediately diluted (~ 10-fold) into an activity assay containing an excess of substrate (250 μM, Km = 60 μM). k_off _was then calculated by fitting the subsequent time course with the equation:

(5) $$P=v_{s}t+\frac{\left(v_{i}-v_{s}\right)}{k_{\text{off}}}\left(1-\text{exp}(-k_{\text{off}}t\right)$$

Inhibition of DPP4 cleavage of active GLP-1 by human DPP4

An LC/MS/MS assay was used to measure GLP-1 cleavage in vitro at room temperature. To limit losses of cleaved GLP-1 (GLP-1_9-36_) due to aggregation and/or adsorption to the walls of the vial, samples were diluted to 40% acetonitrile composition. To determine Km, various concentrations of active GLP-1 (GLP-1_7-36_; concentration range 10 to 240 μM) were incubated with DPP4 (2 nM) in 20 mM phosphate buffer, pH 7.4. Total reaction volume was 125 μl. Every 5 minutes, 28 μl samples were withdrawn and quenched with 28 μl stop buffer (80% acetonitrile, 18% water, 2% formic acid). To determine Ki, two concentrations of active GLP-1 (10 and 50 μM) were incubated with DPP4 (0.45 nM) in 20 mM phosphate buffer, pH 7.4. Total reaction volume was 40 μl. After 90 min, 40 μl stop buffer was added to quench the reaction.

A Thermo Fisher LTQ Orbitrap mass spectrometer in positive electrospray mode was used to detect GLP-1 _9-36_. The chromatography system was a Waters Acquity UPLC with a Waters UPLC BEH C18, 1.7 μm, 2.1 × 50 mm column. A short gradient was employed (0% B to 100% B in 4 minutes) using 98/2 water/acetonitrile (10 mM ammonium acetate) as solvent A and 2/98 water/acetonitrile (10 mM ammonium acetate) as solvent B. A flow rate of 0.8 ml/min was used and 5 μl were injected onto the column. Under these conditions, GLP-1_9-36 _was chromatographically separated from GLP-1_7-36_. GLP-1_9-36 _quantitation was performed by integrating the sum of the 2+, 3+ and 4+ charged ions. Concentrations of GLP-1_9-36 _were calculated from a 1/*x*^2 ^quadratic fit calibration curve (10 nM - 2,000 nM).

### Cynomolgus monkey ex vivo plasma DPP inhibition assay

Male and female cynomolgus monkeys (Charles River BRF, Houston, TX), were housed in an AAALAC-accredited facility (on a 12-hour light/dark cycle) in stainless-steel cages. They had access to purified tap water ad libitum and were fed Harlan Diet 2050 C (Certified 20% protein primate diet). Six monkeys per dose group (3 to 4 males and 2 to 3 females per group) were administered single oral gavage doses of saxagliptin (0.1, 0.3, 1, 3, or 10 mg/kg), sitagliptin (0.3, 1, 3, 10, or 40 mg/kg), or vehicle alone (water, final pH 5.0) at a dose volume of 2 mL/kg. Doses were administered once weekly using a crossover design such that no monkeys received the same dose twice. For the ex vivo cynomolgus monkey assays, blood samples (approximately 0.5 ml) were collected prior to dosing and 1, 2, 4, 6, 8, and 24 hours post dosing in tubes containing K_2_EDTA. Tubes were inverted several times to ensure mixing, and placed on ice. Samples were centrifuged at 5000 rpm for 10 minutes at 4°C for plasma isolation. Plasma was stored at -20°C until analyzed for DPP activity. No difference in DPP inhibition was noted between males and females and so data were combined for analyses.

### Ala-pro-AFC plasma DPP inhibition assay

For the ex vivo assays, plasma from cynomolgus monkeys was diluted with buffer alone. For the in vitro assays, plasma 'spiked' with compound to be tested (10 μl) was added to buffer alone or buffer with drug 'spiked' at concentrations that would yield the same concentration as the 'spiked' plasma in the well (10 μl), followed by the substrate (10 μl, final concentrations were ala-pro-AFC 370 μM and DMSO 3%). This procedure produced a 3-fold dilution of plasma. The buffer contained (final concentration) 33 mM HEPES and 140 mM NaCl (no BSA) at pH 7.9, and the assay was run at room temperature (22°C). A 384-well microplate format was employed to measure plasma dipeptidyl peptidase activity. Activity, the rate of release of fluorescence from the pseudo-substrate, was assayed using a Fluoroskan Ascent microplate reader with rate-determining software. Readings were taken after a 10 second shaking step, at 405 nm excitation and 510 nm emission, with subsequent reads every 1 minute for 18 minutes. Rates were determined from the slope of the regression line fitted to the linear portion of the data (a minimum of 8 minutes and a maximum of 12 minutes from the start of the experiment) showing the increase in fluorescence due to cleavage of substrate over time. Results were expressed as arbitrary units because standard AFC curves were not run for all experiments. The extent of DPP inhibition, derived from the reduction in the initial rate observed at each drug concentration with respect to untreated plasma, were determined and IC_50 _values were calculated using XLfit.

### Gly-pro-pNA Plasma DPP inhibition assay

This assay was reported by Merck as used in their clinical trials [24], using gly-pro-pNA as substrate. The buffer contained 100 mM HEPES with 0.1 mg/ml BSA and the assay was run at 30°C in 96-well microplate format (Costar 9017, Corning. Corning, NY). For the ex vivo assays, plasma from cynomolgus monkeys was diluted with buffer alone. For the in vitro assays, 'spiked' plasma (40 μl) was added to buffer alone or buffer with drug 'spiked' at concentrations that would yield the same concentration as the 'spiked' plasma in the well (40 μl), followed by the substrate (20 μl, final concentration of gly-pro-pNA is 400 μM). Activity, the rate of release of free pNA from the pseudo-substrate, was assayed at 30°C, using a Spectramax 340 plate reader (Molecular Probes) with rate determining software. Absorbance at 390 nm was measured. Results were expressed as arbitrary units because standard pNA curves were not run for all experiments. The extent of DPP inhibition and IC_50 _values were calculated as described above.

### Statistical analysis

The SAS JMP statistical package was used for all statistical analyses. A Dunnett's test was used for comparison to vehicle and all pairs were compared with a Tukey HSD.

## Abbreviations

DPP: Dipeptidylpeptidase. (1*S*,3*S*,5*S*)-2-[(2*S*)-2-Amino-2-(3-hydroxytricyclo[3.3.1.1^3,7^]dec-1-yl)acetyl]-2-azabicyclo[3.1.0]hexane-3-carbonitrile, monohydrate, Saxagliptin, Onglyza^© ^or BMS-477118; GLP-1: Glucagon-like peptide-1.

## Competing interests

All the authors were employees of Bristol-Myers Squibb at the time that this work was performed.

## Authors' contributions

AW carried out some of the in vitro enzyme measurements and the ex vivo assays and contributed to writing the manuscript. CD carried out some of the in vitro enzyme measurements and the ex vivo assays and contributed to writing the manuscript. LK carried out some of the in vitro time-dependence assays. GL carried out some of the in vitro time-dependence assays. RL measured GLP-1 concentrations and contributed to writing the manuscript. EH conceived and ran the cynomolgus monkey studies and contributed to writing the manuscript. PS conceived the GLP-1 assay. JM conceived and designed the in vitro time-dependence assays and contributed to writing the manuscript. LH conceived and designed the compounds, some of the in vitro assays and contributed to writing the manuscript. MK conceived and designed these studies, coordinated the work and was the primary author of the manuscript. All authors read and approved the final version of the manuscript.
